# Purging the Judiciary After a Transition: Between a Rock and a Hard Place

**DOI:** 10.1007/s40803-024-00201-y

**Published:** 2024-03-04

**Authors:** Katarína Šipulová, David Kosař

**Affiliations:** 1 Judicial Studies Institute, Faculty of Law, Masaryk University, Brno, Czech Republic; 2 Department of Constitutional Law and Political Science, Faculty of Law, Masaryk University, Brno, Czech Republic

**Keywords:** Transitional justice, Lustration, Purging, Judiciaries, Judges, Judicial turnover, Removal of judges, Central and Eastern Europe

## Abstract

Judges play a key role in the implementation of transitional justice mechanisms. Yet, less attention has been paid so far to the question of how to address their collaboration with non-democratic regimes. In theory, judges can be subjected to virtually all transitional justice mechanisms ranging from criminal prosecution and lustration to truth-seeking, or even amnesties. However, we show in a case study of Czechia that these mechanisms are not well equipped to address the complicity of judges in past crimes for three reasons: (1) judges usually play different roles in past crimes from political elites, (2) the principles of the separation of powers and judicial independence preclude the easy replacement of judges, and (3) pragmatic exigencies, such as the shortage of lawyers who are not tainted by cooperation with the previous regime, further complicate the renewal of the bench. Nevertheless, we argue that the lack of recognition of the role judges have played in non-democratic regimes is dangerous, as it may negatively affect public confidence in the judiciary and taint its legitimacy. Examples from Hungary, Poland and Romania, moreover, show that populist leaders are tempted to abuse the transitional justice rhetoric use the failure to deal with the past of judges as a justification for their court-curbing practices. Post-transition purges are therefore stuck between a rock (interfering in judicial independence and practical exigencies) and a hard place (mental dependence of the judiciary on the previous regime, low public trust in courts). When the democratic opposition defeats the populist leader, such as in Poland in 2023, it unfortunately faces the same dilemma. Thus, the Czech way of dealing with the past within the judiciary in transition from communism to democracy (transition 1.0) provides important insights also for today’s undoing of populist judicial reforms and transition from authoritarian populism to democracy (transition 2.0).

## Introduction: Revival of Transitional Justice Debates

In August 2023, the former vice-president of the International Criminal Court, Robert Fremr, withdrew his candidacy for the position of judge of the Czech Constitutional Court. With his impressive international experience,^1^ Fremr was meant to be one of the key candidates in the newly reformed process of appointment of constitutional justices, which highlighted the need for a transparent, merit-based selection that would secure a better quality as well as greater diversity of candidates.

Fremr’s resignation arrived in response to the intense political and medial backlash^2^ after the Czech Institute for the Study of Totalitarian Regimes publicised information that Fremr, who had served as a criminal judge under the communist regime between 1982 and 1989, had participated in a political trial and, moreover, sentenced 166 individuals to prison in their absence for illegal emigration from communist Czechoslovakia.^3^ The public was immediately swept into a highly polarised discussion. How could communist-era judges who actively participated in or legitimised human rights violations still enjoy a place among the democratic elite? Were lustration and vetting processes not successful? Should Fremr’s failure to revolt against the communist law disqualify him from holding a position as a constitutional judge, or did he manage to rehabilitate his profile and regain credibility through his 30-year-long career in international criminal law arena?

There are two interesting dimensions to this story. First, Czechia has long been praised as a poster child for transitional justice and de-communisation. The Czech lustration law that aimed to remove old communist cadres from public life has been referred to as ‘thorough and comprehensive,’^4^ ‘one of the strongest’,^5^ and even ‘the most sweeping’^6^ among the post-communist countries of Central and Eastern Europe (‘CEE’). It intentionally applied also to judges as the courts played an important role in upholding the legitimacy and strength of the communist regime.^7^ Yet, Fremr’s story is not the first in which the communist past of judges has returned to public discussion and prompted analyses of why the (allegedly vast and thorough) processes of transitional justice failed to de-communise the Czech judiciary.

Second, this reflection on the shortcomings of the de-communisation of judiciaries resurfaced right at the moment when the transitional justice narrative returned to Europe. The increasing intensity of a political backlash against the courts documented in Poland, Hungary, Romania and Israel has reminded us how much undemocratic regimes care about the capture of an independent judiciary and how important a role packed judges play in the implementation of their, often unconstitutional, policies.^8^ The liberal opposition’s success in the 2023 Polish parliamentary elections highlighted the urgency of resolving the question of what to do with unlawfully appointed judges,^9^ or how to un-pack the captured courts.^10^

Democratic elites returning to power after periods of backsliding face no easy dilemma. Should they try to remove all illegitimately appointed judges, irrespective of their behaviour? Or, instead, should they aim to ‘sift out the bad apples’ and remove those judges that appear to be loyal to the previous elite? Can they limit the cleansing at apex courts? And how can these removals be done without imposing further strains on principles of the rule of law?

While addressing these questions, many scholars ask to what extent we can rely on transitional rule of law^11^ and the toolbox of transitional justice,^12^ and how to make mechanisms of transitional justice compatible with commitments to international human rights law^13^ and requirements laid down by European supranational courts.^14^

The dilemmas outlined above remind one of the core discussions led by CEE democratic elites in the early 1990s. Terms like vetting and lustration returned to public and scholarly discourse. Yet, scholars also repeatedly pointed out that, despite the generally rich literature on how countries come to terms with the crimes of the past,^15^ why they chose to forgive or prosecute the perpetrators,^16^ or how effective vetting and lustration of political elites was,^17^ only limited attention had been paid to purges of judges.

This gap in the transitional justice field is surprising. First, judges played an indispensable role in communist regimes. Communist parties kept judges on a short leash, instructed them how to decide politically salient cases (a practice colloquially referred to as ‘telephone justice’^18^), or used them to consolidate and display the parties’ power in political show trials.^19^ Second, courts, often staffed with the very same judges, later on played a crucial role in the application of the transitional justice policies of young democratic regimes.^20^ They reshaped many political agreements on transitional justice, implemented victim-oriented programmes and addressed doctrinal clashes between principles of transitional justice and newly reinforced commitments to fundamental rights.^21^

Understanding the logic and effects of judicial purges is even more important in the face of the first empirical studies from Latin America which suggest that the totalitarian past of judges has consequences for their decision-making during the transition. For example, judges who participated in or condoned past crimes are less likely actively to approach transitional justice issues,^22^ or are even more willing to block them.^23^ While some studies demonstrate that judges may support transitional justice in order to atone for their role in past crimes,^24^ others hypothesise that the successful implementation of transitional justice decisions depends on judicial reform and the commitment of judges to the rule of law.^25^ Despite of this significance, the greater part of the relevant scholarship has perceived judges as second-order actors of transition, and paid almost no attention to cleansing within the judiciary itself.

In this article we step into this field and argue that the historical analysis of effects and forms of judicial purges after 1989 can bring important insights into current CEE discussions on how to restore judicial independence and undo court-packing reforms introduced by populist governments. We use the study of how Czechia came to terms with the totalitarian past of judges to demonstrate two critical points. First, the Czech story uncovers the limits of transitional justice tools when applied to judiciaries. The post-1989 de-communisation of the courts consisted of five different mechanisms aimed at cleansing the courts of communist cadres, from general measures like lustration to tools specifically tailored to the judiciary (like retention elections or the removal of court presidents). However, the majority of these were not successful. They clashed with principles of judicial independence, as well as with practical exigencies such as a shortage of new judges or educated lawyers willing to join the judiciary.

Second, the Czech experience provides important lessons for those countries, that aim to restore their judiciaries after court-packing and democratic backsliding, like Poland, or potentially Hungary and Israel in the future, or EU candidate countries that repeatedly attempt to tackle widespread judicial corruption and low public trust in courts, like Georgia and Albania. On the one hand, purges of judges appointed by populist leaders are problematic for both theoretical and pragmatic reasons as they encroach on the principle of judicial independence and, particularly in smaller countries, are difficult to carry out due to the lack of new qualified judges. On the other hand, the decision to bury the past and leave the illegitimate selection or biased behaviour of judges unaddressed endangers not only the implementation of transitional justice, but also the development of the rule of law and judicial independence in general. Moreover, the experience from CEE suggests that in order to be effective, transitional justice must be visible. Otherwise, non-democratic political leaders are quick to exploit the opaque past of judges to delegitimise them in the eyes of the public and to justify their own future court-packing policies.^26^

The article proceeds as follows. Section 2 briefly introduces the communist capture of the judiciary. Section 3 discusses the transition of the Czech judiciary and explains the logic of five different mechanisms aimed at judicial cleansing. Section 4 analyses the effectiveness of individual measures and problematises their impact on judicial independence. Section 5 juxtaposes this historical experience with current efforts at judicial purges. Section 6 concludes.

## The Role of the Judiciary Under the Communist Regime

After the fall of the Austro-Hungarian Empire in 1918, the new democratic Czechoslovakia kept a bureaucratic career model of the judiciary, a system of court administration run by the Ministry of Justice and the hierarchical ideal of officialdom.^27^ However, the February 1948 communist coup d’état revamped the Czechoslovak judicial system. After the coup d’état, the Czechoslovak communist regime quickly remodelled the judicial system and suppressed judicial independence. Once the Communist Party had realised that the original Marxist prophecy of the state and law ‘withering away’ was not about to materialise, the law became critical in preserving communist power.^28^ Therefore, in contrast to those authoritarian regimes which attempted to insulate the courts from politics,^29^ judges became an important weapon of the Czechoslovak Communist Party.

In the first years after the coup, the Communist Party abolished all courts that could scrutinise its work^30^ and adopted a grand-scale court-packing plan. Since only very few professional judges joined the Communist party immediately after 1949, it introduced an institute of lay judges. These were pre-screened by the Party and were in the majority in all tiers of the general courts including the Supreme Court.^31^ Moreover, the Party created an extraordinary court (the State Court) that dealt with show trials of political opponents,^32^ and it installed trusted loyal communists as judges of the Supreme Court and presidents of the general courts.^33^ Furthermore, through the so-called ‘security fives’ (*bezpečnostní pětky*),^34^ the Communist Party instructed judges how to decide cases that were sensitive for the communist regime, imprisoned those few ‘recalcitrant’ judges who dared to disobey Party orders, and introduced a Soviet-style *prokuratura* which exercised tight oversight over judges.^35^

Once the Czechoslovak communist regime had ensured that it had full control over the judiciary, the Party abolished the State Court, gradually reduced the role of lay judges, and left judges some autonomy in deciding civil and ordinary criminal cases.^36^ However, it kept judges on a short leash through the ‘body that elects judges can also dismiss them’ principle and a system of regular retention elections.^37^ This allowed the Communist Party to get rid of problematic judges without attracting any public attention.

The 1968 Prague Spring movement reformed this system. However, the Communist Party quickly re-established its control after the Prague Spring movement was crushed. It relied primarily on regular retention elections, the specific retention of all judges who reached the age of 65, and the expulsion of judges who, by actively participating in the Prague Spring, had violated their judicial oath by ‘betraying the working class’.^38^ Resistance by the bench was almost non-existent.^39^

1989 therefore found the Czechoslovak judiciary as a subservient branch with little independence and prestige. Public confidence polls conducted in 1990 showed that only 28 per cent of citizens trusted Czech courts.^40^ Moreover, the purges after the crushing of the Prague Spring not only secured fast ‘normalisation’, but also created a specific mindset among Czech judges that could not easily be changed with the imposition of the new formal institutions.^41^

The transitional processes aimed at communist courts had to tackle two different dilemmas. The first dilemma was: How to deal with communist-era judges who participated in violations of individual rights? Post-communist courts in 1989 still contained a small number of judges who had been involved in political show trials (the most gruesome processes, however, dated back to the 1950s), judges who had penalised actors in 1968 Prague Spring, as well as judges who, in line with communist law, had imposed sanctions on those individuals who had illegally emigrated from Czechoslovakia, or had spread pro-Western propaganda or were otherwise involved in ‘anti-state activity’. Military, criminal and labour courts in particular were full of judges who had served as the right hand of the Communist party.

The second dilemma was: How to deal with judges appointed by the communist regime, who did *not* participate in any politicised trials or human rights violations, but who were loyal to the ideology of the Communist Party, or who shared the professional role conception of judges in the political system created and nurtured by the Communist Party.

These two dilemmas point to two separate categories of judges and trigger different rationales for their potential purges: (I) those who actively participated in violations of human rights; and (II) those who were ideologically aligned with the previous regime (Table 1). Transitional judicial purges of these two categories of judges were built on four different normative justifications. First, the removal of judges who actively participated in violations of individual rights (1) belongs under the core umbrella of transitional justice. The source of judges’ accountability is two-fold. It targets either those judges who explicitly violated domestic communist law (e.g. by taking part in politicised processes and show trials, manipulating evidence or engaging in corruption), or those judges who acted in line with domestic legal provisions, but in doing so violated norms or general principles of international human rights law. Individual accountability based on principles and norms of international law, however, requires these norms to be effective and directly applicable by the judges in question (for more on this see Sect. 3 b). Otherwise, the removal of judges who acted against international law might be seen as contrary to the rule of law and in violation of both the principle of non-retroactivity and of judicial independence.^42^

**Table 1:** Conceptualisation of judicial turnovers after the transition. *Source*: Authors

Category of judges	Justifications		TJ toolbox
**I. PERPETRATORS OF HR VIOLATIONS**	**1. Individual accountability of judges**	Violation of domestic law (political trials, corruption)	Core TJ processes
		Violation of international HR la (political prisoners, punishment of emigration, …)	
**II. IDEOLOGICALLY ALLIGEND OR INDOCTRINATED JUDGES**	**2. Indoctrination of judges (moral and ethical standards)**		Derived TJ processes
	**3. Lack of expertise**		
	**4. Illegitimate appointment**		

The purges of indoctrinated judges sought to remove them because of their indoctrination (2), lack of expertise or ethical standards (3), and the illegitimacy of their appointment^43^ (4). While these processes are not aimed at the removal of perpetrators of crimes, they seek to reform those structures of a political system that failed to prevent crimes from happening. Because of their loser link to normative aims of transitional justice, we label them as derived transitional justice processes.

It is also worth noting that this second category of purges resulted from the implementation of core transitional justice tools used in CEE de-communisation processes, such as lustration and vetting in general. It also corresponds to the recent trend which seeks to enlarge the legitimacy of judicial vetting from transitional justice to other reasons such as large-scale corruption (Albania in 2017, Ukraine in 2014, or more recent debates in Macedonia, Georgia and Kosovo). Compared to the first category, derived TJ processes do not require individual accountability or intentional violation of individual rights on the part of the judge.

Against this conceptual backdrop, we next identify all mechanisms adopted to reckon with the communist past of the Czech judiciary and analyse their effects.

## Reckoning with the Communist Past within the Czech Judiciary

The early post-1989 transitional justice debates addressed the role of communist-era judges in the new democratic judiciary, although admittedly with much less vigour and publicity than lustration targeting politicians. The moral dilemmas related to judicial purges were very similar to those accompanying the vetting of party officials, executives or members of the armed forces.^44^ They focused mainly on the active role played by judges in violations of human rights committed between 1948 and 1989. The political discussion on removals of judges did not stir up as many controversies and was, more or less, a derivate of the general decision on how to deal with past elites. The extent to which individual judges had participated on human rights violations was left for the consideration of the courts and the prosecution in individual proceedings.

However, the new democratic actors also understood that the removal of “the judges-perpetrators” (first category of removals) would not be enough to redeem the poor reputation of the judiciary in Czech society and that the renewal of public trust in the courts would require a systemic addressing of the past among all sitting judges (second category of removals). The courts were absolutely vital to the success of the government's efforts to implement restorative processes (rehabilitation of victims and restitutions of property) and to establish a new legal framework necessary for the economic reform of the country. Moreover, the government also needed to create a hitherto missing branch of administrative courts and fill hundreds of new judicial positions.^45^

The Federal government therefore decided that the new culture of the rule of law would rest on two pillars: (1) cleansing the courts of the communist legacy, and (2) increasing the prestige of judges.^46^ The communist regime left the judiciary extremely weak. As explained in the previous Section, the pre-1989 courts were under the tight control of the Communist Party, executed via a triad of the ministry of justice, the general prosecutor and court presidents. The Communist government packed the courts with judges who were willing to comply with the political interpretation of law. Some were by conviction hard-core communists, while others complied with the communist expectations for prudential or career reasons. Some judges even entered the judicial system after a very questionable and speedy legal education—sometimes lasting no longer than a year. This combination of poor expertise and formalistic decision-making resulted in the very low prestige of and public confidence in the courts.^47^ Judicial purges advocated by the new government therefore wrestled with a dual task: to get rid of judges compromised by their communist past and of judges of poor quality and ethical standards.^48^

The dilemma before the new democratic politicians of 1989 was hence very similar to the current discussions in Poland.^49^ They wanted to get rid of judges who had actually collaborated, as well as of judges who were perceived by the public as loyal to the previous regime. At the same time, politicians did not want to erode judicial independence any more than necessary. Moreover, they could not afford to dismiss too many judges because, ‘[i]n contrast to East Germany, there was no West Czechoslovakia … which might have easily … re-staffed judicial posts virtually overnight.’^50^ Not surprisingly, the negative image of the courts, mediocre working conditions and poor salaries did not attract many new candidates into the judiciary. This fact significantly tied the hands of the post-1989 government.

Despite this dilemma, Czechia adopted an impressive number of transitional justice mechanisms aimed at judicial purges. Apart from the best known (1) lustration of judges, Czech political leaders actually implemented four additional mechanisms: (2) removal of the communist-era court presidents; (3) retention elections for communist rank-and-file judges; (4) the disciplining of communist-era judges for dereliction of judicial duty; and (5) the criminal prosecution of communist judges for the most egregious violations of judicial duty. In what follows we introduce the logic and expectation behind each of these mechanisms in chronological order.

### Five Mechanisms of Judicial Purges

In the first 3 months of 1990, the Minister of Justice started with a radical transitional justice step and in a large-scale political decision removed 65 court presidents and vice-presidents.^51^ This meant that virtually all *court presidents were replaced*. Given the fact that court presidents were ‘the most reliable cadres [who] not only implemented party resolutions, but, in practice, ruled over the judges and personified the dictatorship and the subordination of the system of justice’,^52^ this was an important step in the de-communisation of the Czech judiciary.

Despite this effort, on 30 March 1990 Czech MPs criticised the Minister of Justice for insufficient de-communisation of the judiciary. The *retention elections* of judges that took place at this meeting led to a heated debate, during which deputies raised objections to proposed candidates and questioned their past. Nevertheless, judges managed to persuade MPs that they were just small cogs in the wheel of the Communist regime who had prevented the worst from happening. This controversial narrative eventually prevailed, and the candidates for whom the Minister of Justice had provided a personal guarantee were re-elected, including those who had participated in the conviction of well-known political prisoners.^53^ This resulted from a disagreement on how far the individualised guilt of communist-era judges should go, and whether decision-making in line with communist-era laws can be considered so heinous a crime that it required removal. Overall, the retention election had limited link to individual accountability. Instead, it focused much more on the renewal of formal legitimacy of those judges who were reappointed by the democratic regime.

The attempts to purge the judiciary via retention extended far beyond 1990. The new 1991 Law on Courts and Judges, which entered into force on 1 September 1991, stipulated that all judges elected before 1 January 1990 (i.e. during the communist era) had to be reappointed under the new rules within 12 months. As a result, eight Federal Supreme Court judges had lost their jobs by August 1992 due to retention legislation.^54^ These, together with five judges who were leaving the court as a result of lustration, left the Federal Supreme Court, which adjudicated in panels of five judges, on the brink of dysfunctionality, because only four judges in the Civil Section, three in the Criminal Section, three in the Commercial Section, and six in the Military Section remained.

However, the high turnover of judges at the Federal Supreme Court was an exception. Overall, almost all Czech judges targeted by retention were eventually re-appointed. This was in stark contrast to the way reunified Germany addressed the reckoning with the past in the judicial sector. The German authorities adopted a similar scheme, under which all active judges in the German Democratic Republic had to re-apply for their jobs. They had to fill out a complex questionnaire and their applications were scrupulously screened.^55^ As a result, only 10 per cent of former GDR judges were reappointed in Berlin. The average success rate in other East German states was higher (55 per cent), but these purges were still very thorough.^56^

The 1991 Czech Law on Courts and Judges also introduced the third mechanism for reckoning with the problematic past of communist-era judges, the *disciplinary liability of judges for dereliction of judicial duty* during the communist regime. More specifically, it empowered the disciplinary courts to dismiss judges for wrongdoing during that era. These measures provided clear grounds for holding communist-era judges to account for their past behaviour.^57^

The Czech Ministers of Justice initiated several disciplinary motions in which they tried to invoke these provisions in order to get rid of the judges with the most problematic past, who sentenced dissidents to imprisonment for the exercise of their freedom of political speech and the publication and distribution of the Charter 77 document during the late 1960s. However, the Czech disciplinary courts took a protective stance and required evidence of repeated instances in which an impugned judge had interpreted the law in an excessive and politically tendentious fashion.^58^

An illustrative example is the decision of 31 August 1994 in which the Supreme Court acquitted an ex-communist judge who had convicted two individuals for dissemination of anti-state publications (mostly Charter 77). The Supreme Court’s reasoning perhaps best interprets the deeply rooted formalism and judicial solidarity. When the Minister of Justice argued in his disciplinary motion that the given judge had violated the International Covenant on Civil and Political Rights (ICCPR), ratified by communist Czechoslovakia in 1968, the Supreme Court simply denied the direct effect of the ICCPR. The Supreme Court stressed that ‘…as follows from legal theory and practice, the international treaty did not have primacy over domestic legal statute [under communist law]…Until then [meaning until the new Constitutional Law 23/1991], the state merely had an obligation to secure the conformity of its legal provisions and statutes with the international treaty.’ In other words, any potential violation was a problem for the state administration, not the courts, which decided the case according to the domestic legal provisions. Thus, the Supreme Court was willing to assert that during the communist era the ICCPR was no more than an ethical commitment.

Only after these events, in October 1991, did the Federal Assembly pass the *Large Lustration Law*. Three factors raised the stakes and the interest of the new Czech elites in lustration: (1) the fear of ‘skeletons in the closet,’ and the insecurity of political leaders about which of their party members had a communist past giving rise to the risk of future exposure or blackmail;^59^ (2) the 1990 scandal when 15,000 secret police documents disappeared from the archives;^60^ and (3) the surprisingly positive result for the Communist party in the 1990 elections. The tacit agreement established between the communist elites and dissidents at the roundtable talks slowly dissolved and the need for a lustration law became apparent.

Thus, in October 1991 Czechoslovakia adopted the first and strictest lustration law in the CEE region, the so-called Large^61^ Lustration Law.^62^ The parliamentary debate on the Lustration Law was extremely heated and, once adopted, the Law was severely criticised for its breadth by academics and international institutions,^63^ and also by many dissidents.^64^ The part of it that dealt with citizens’ collaboration with the secret police was the most controversial,^65^ as the law affected not only agents, informers and political collaborators, but also candidates for collaboration. The Venice Commission’s stance on lustration was discussed during the parliamentary debate, but not taken seriously. The MPs identified lustration as a necessity for personnel discontinuity and the establishment of minimal justice.^66^

The Lustration Law envisaged the preparation of two lists: the so-called ‘protected positions’ and the ‘suspect positions’ lists.^67^ The list of protected positions referred to those offices and positions appointment to which requires a negative lustration certificate (indicating no involvement with the communist regime). The ‘protected positions’ include *inter alia* all those filled by election, nomination or appointment to bodies of state administration, the army, the security service, the police force, staff working in the offices of the President, Government, Parliament, the courts, state radio and television, and state-owned companies.^68^ The second list, suspect positions, covered offices held or activities done during the communist regime that disqualified their holders from working in capacities included in the first list of ‘protected positions.’ Thus, people who fell into one of the categories in the list of ‘suspect positions’ were barred from holding ‘protected positions’.

The law, which is still in force, thus does not affect communist party members in general. In practice, it has affected mainly the state security forces, army, secret police collaborators, higher communist party officials, members of the people’s militia and the purge committee. The law targeted approximately 400,000 people.^69^ Individuals who received ‘positive lustration’ in the screening (i.e., they were proven to have collaborated or to have held one of the proscribed posts) could still participate in political life, as the law did not limit their active or passive suffrage. The law was originally meant to remain in force for five years but, despite two challenges before the Czech Constitutional Court,^70^ it is still in force.

This Law applied to judges, among other public figures. As a result, a judge who in the communist era had held one of the ‘suspect positions’ outlined above could no longer remain in office. It was up to court presidents to require negative lustration certificates from the judges at their courts. This regulation had certain flaws though. In particular, many apex court judges refused to submit certificates or any other information on their communist pasts. They claimed that lustration laws violated the ICCPR and the Czech Charter of Fundamental Rights and Freedoms. In total, next to five judges who were removed due to positive lustration findings, seven Czech Supreme Court judges refused to submit lustration certificates or submitted incomplete information. As a result, the Minister of Justice initiated disciplinary motions against them. Nevertheless, the division of Czechoslovakia prevented the completion of these proceedings.^71^

Finally, Czechia also suspended statutory time limitations for judicial murders and paved the way for the *criminal prosecution* of judges for the most egregious violations of judicial duty that had taken place during the show trials in the 1950s. Nevertheless, no criminal prosecutions were initiated against professional communist-era judges in Czechia. In the end, the only judge who was successfully prosecuted was a lay judge, Ludmila Brožová-Polednová, who had taken part in the show trial of Milada Horáková, an opposition politician and former prisoner in Nazi concentration camps.^72^ Brožová-Polednová was charged with being a prosecutor in this political trial and accessory to murder. She was found guilty of assisting in judicial murder and sentenced to 8 years in prison. The Czech courts carefully defined both the concept of judicial murder and the role of judges in committing it.^73^ However, the symbolism of this case was watered down due to Brožová-Polednová’s age. In 2009, she was officially the oldest Czech prisoner (being 86 at the time of the final verdict) and President Václav Klaus pardoned her just a year later.

All in all, as we will demonstrate in the following Section, only a small number of judges have been prosecuted for the abuse of their power or the violation of individual rights under the communist regime.^74^

## The Aims and Effects of Czech Judicial Purges: Bold on Paper, Meagre in Practice

Generally speaking, the purges within the CEE post-communist judiciaries were rather minimal. For example, in Romania ‘a large proportion of the ordinary judges … held their former public offices during the 1990s, even if they had been appointed during the previous regime.’^75^ The situation in Bulgaria,^76^ Hungary^77^ and Slovakia^78^ was very similar. Only Poland did slightly better, as it purged at least the Polish Supreme Court.^79^

Although Czechia is often presented as an outlier among the CEE countries^80^ with relatively complex vetting programmes, the number of those who actually left the bench was rather low. Between January 1990 and December 1992, approximately one third, i.e. 484 out of 1460 communist-era judges, left their jobs.^81^ Moreover, the majority of these judges were dismissed for a reason other than lustration, or left their posts voluntarily in order to improve their financial situation in the quickly developing private sector.^82^

In what follows we offer a bird’s eye view of how all five identified mechanisms of judicial turnover worked in practice. We start with an outline of the aims of political actors associated with individual mechanisms and then discuss their effectiveness.

### Expectations

As already mentioned, the discussions on judicial purges addressed both categories of judges: those who actively participated in violations of individual rights, and those whose legitimacy was compromised simply by the function of courts under the previous regime. The adopted mechanisms of judicial purges explicitly invoked three out of four possible aims (Table 2): (1) the individual accountability of judges for communist crimes (which corresponds to the core transitional justice narrative), (2) the moral and ethical standards of communist-era judges, and (3) their expertise and education (both reflecting the *derived transitional justice dilemmas,* which were specific to the judiciar*y* and did not appear in other transitional justice areas).^83^ One remaining issue that was not mentioned by the incoming political elite was the potential delegitimisation of past appointments made by the communist power. This was understandable, as the court-packing and other forms of selection of judges that contravened the law and constitutional principles had happened decades ago and their effects had already been diluted by time. Therefore, the education, expertise, and ethical standards became secondary considerations.

**Table 2:** Overview of the effectiveness of and opposition to individual mechanisms aimed at purging the Czech judiciary. *Source*: Authors

Mechanism	Declared aim	Opposed by	Success	Reason
**Lustration**	1. Aim: Individual accountability (category I)	Communists, court presidents, Supreme Court judges	Partial	Understaffed courts
				Lack of data
				Many judges left on their own
**Criminal prosecution**	1. Aim: individual accountability (category I)	Judges, prosecutors	No	Judicial independence
**Removal of court presidents**	1. Aim: individual accountability (category I)	Communist-era court presidents	Yes	Pure transitional political decision
	2. Aim: Ethical standards (category II)			
	3. Aim: Expertise (category II)			
**Disciplinary liability**	1. Aim: individual accountability (category I)	Judges, court presidents	No	Non-retroactivity principle
	2. Aim: Ethical standards (category II)			Judicial solidarity
	3. Aim: Expertise (category II)			Many judges left on their own
**Retention election**	1. Aim: individual accountability (category I)	Court presidents (communist-era and new democratic), judges	Partial	Judicial independence
	2. Aim: Ethical standards (category II)			Judicial solidarity
	3. Aim: Expertise (category II)			

The focus on expertise and moral integrity stemmed from the instrumental use of courts by the communist regime. Communists put their idea of the popularisation of the judiciary into practice by staffing it with lay judges who lacked university or high school education, and who had gained the necessary qualification in fast-track legal courses set up by the regime.^84^ For professional judges, the requirement to have a university law degree was dropped soon after the 1948 communist coup d’état and re-established only in 1964. The career model of the judiciary inherited from the Austrian Empire and the interwar period, when new candidates entered the ranks of the judiciary in their early twenties, combined with political elections overseen by the Communist Party, socialisation within the judiciary, and dependence on older peers prevented there being a significant increase in the quality of the communist judiciary. Transitional purges therefore had to address all of the complicity of judges in the communist regime, the deeply ingrained logic of judicial dependence, and the lack of ethical and intellectual standards.

### Outcomes of Judicial Purges

How successful were individual mechanisms? Table 2 demonstrates also the results of our analysis, and for each mechanism identifies the officially declared aim, who was most critical of its implementation, and the evaluation of the effect—how successful the mechanism was and why.

Given the prominent role of lustration within Czech transitional justice, it comes as no surprise that lustration also attracted the most attention among mechanisms aimed at judicial cleansing. Political elites saw lustration as a means to remove judges who participated in violations of individual rights (including those guaranteed by supranational human rights commitments). The trust vested in lustration as the most appropriate mechanism was strengthen by the Supreme Court and the Office of the General Prosecutor,^85^ who jointly advocated the incompatibility of function as a judge with active collaboration with former secret police as the core aim to be achieved in the judicial turnover.^86^

All sitting and newly appointed judges had to be screened for negative lustration. The results of lustration triggered political and public interest, especially in the selection of apex court judges.^87^ The biggest impact of lustration was in the case of the military courts (abolished in 1993), where lustration managed to filter out those who had held important posts in the Communist Party’s organisation.^88^

Yet, the overall effect of lustration in the judiciary was much lower than scholarship had hypothesised.^89^ The reasons are several. By November 1991, when the Large Lustration Law came into effect, the majority of the ‘compromised judges’ had already left the judiciary. Lustration therefore arrived in an atmosphere of understaffed courts frantically seeking new candidates for vacant or new judicial posts. The critical shortage of judges meant that newer judicial positions were once again filled by judges from the communist era.

The implementation of lustration was also complicated by the specific role of judges in the communist regime. Lustration primarily tackled people who had held high-ranking positions in the Communist Party or who had worked for or cooperated with the secret services. That was highly improbable in the case of judges. Under the career model, judges joined the courts at a very young age, and it was hardly improbable that they would manage to hold important official positions inside the Communist Party. Moreover, they were carefully screened, both when they entered law school and when they were appointed, and thus the communist regime did not need to keep additional tabs on them by forcing them to join the ‘Peoples’ Militia’ or to cooperate with the State Security Police.

At the same time, judges with positive lustration certificates fought back—and often successfully. Several judges pressed charges against the Ministry of the Interior for false and unjustified listing of names in the archive files of the State Security Police and for violating their rights to privacy and human dignity. The general lack of credibility, frequent falsification and fabricated stories in secret archives led courts to quash the majority of the positive lustration certificates relating to both judges and other public servants.^90^ In some cases, the courts found the ministerial decisions too sweeping and poorly justified.^91^ In other cases, listings in secret police archives were questioned for lack of proof beyond reasonable doubt.

All in all, lustration seemed to be a sieve for different grains and failed to capture typical examples of judicial complicity in human rights abuses during the communist era. The belief of the new political elite that lustration would rid the judiciary of judges who committed any violations of individual rights fell short. In theory, lustration of judges could have weakened their ties with political actors and removed those judges who had dangerous informal ties with the political arena, but it arrived too late and did not operate with the understanding of what role judges actually had done under communism.

Criminal prosecutions, like lustration, focused on the first aim and on the individual accountability of judges for human rights violations committed under the communist regime. In theory, criminal prosecutions were better equipped to capture the conduct of compromised communist judges and to force them out of the system. According to the 1991 Law on Courts and Judges, any criminal prosecution required the agreement of the political authority which originally appointed the judge. In the case of communist-era judges, this was the Czech Parliament. As it turned out, however, political agreement was the smallest obstacle to be overcome. While MPs appeared to be eager to purge compromised judges,^92^ the prosecution was not ready to bring enough criminal cases and judges were reluctant to criminalise the behaviour of their peers who followed the letter of the communist Czechoslovak law, but who violated higher constitutional principles or commitments from international human rights law.

Apart from potential hypotheses on judicial solidarity, the couple of cases which did make it to the criminal courts demonstrated the many procedural and legal difficulties in prosecuting communist judges, related mostly to the principle of non-retroactivity. Next to the suspension of statutory time limitations for judicial murders, the only other documented case we found was the criminal prosecution of two judges from the Prague Municipal Court, in which the Federal General Prosecutor managed to prove that they had kept seven people accused of treason (spreading dissident literature transported from France) in detention without any relevant legal cause. Both judges were first dismissed and then, after the approval of the Parliamentary assembly, tried for the abuse of their power.^93^

On the opposite site of the spectrum, the removal of the communist-era court presidents in 1990 worked relatively well. One of the key Czech transitional justice mechanisms aimed at removing those judges who had acted as transmission belts for the Communist Party^94^ and to prevent them from functioning as court presidents,^95^ with a potentially crucial impact on the further selection of rank-and-file judges.^96^ The removal also met with minimal opposition, which secured the Minister of Justice sufficient legitimacy swiftly to carry out the dismissals and fill the posts with new pro-democratic court presidents.

Disciplinary proceedings, like criminal sanctioning, also relied on the already existing concept of judicial accountability. The disciplining of communist judges pursued all three aims of the purges. They targeted both judges who committed violations of individual rights (as already mentioned, mostly by sentencing political opponents and dissenters to prison) and judges who lacked ethical and moral qualities or professional skills and expertise. The legal background for this disciplining appeared in the 1991 Law on Courts and Judges, which contained a specific provision allowing for the dismissal of a judge who had violated his or her obligation and duties, or had otherwise harmed judicial independence between 25 February 1948 and 31 December 1989. In the eyes of the government, this provision was supposed to be another breakthrough in judicial purges, as the Rehabilitation Act allowed criminal prosecution only for explicit material violations, while the majority of communist judicial misdeeds had taken place in the context of interference in judicial decision-making by the public authorities.^97^

The promising mechanisms, however, failed to deliver the goods. First, the Minister of Justice failed to collect sufficient evidence to meet the required standard of proof in disciplinary petitions. Second, of the dozens of cases that were begun, the majority were dismissed by the disciplinary courts. The Supreme Court in particular demonstrated a high level of solidarity with communist-era judges when it stuck to a formalistic interpretation of communist law and refused to implement the protection of individual rights following from the then existing international human rights commitments. The Supreme Court relied on a dualist interpretation of international human rights commitments and argued that the ratification of two international covenants bound only the executive power, not the judiciary. According to the Supreme Court, any other interpretation would put too great of a burden on communist judges, and would punish them with retroactive rules.^98^ The Supreme Court was not able nor willing to solve this dilemma.

Just like disciplining, retention elections were intended to cover all three aims of judicial purges—to remove several generations of dependent judges, tackle the degradation of the role of a judge by raising the requirements on ethical and moral standards and, finally, to strengthen the level of expertise and the intellectual qualities of judges.^99^ The greatest motivation behind the retention election was to secure that judges who had participated in communist justice would not implement the transitional justice policies—especially the large-scale restorative processes.^100^ All retentions were also conditioned by a negative lustration of re-elected judges, but the power of the Minister of Justice not to reappoint a judge was much broader and could rest on the candidate’s inadequate ethical qualities or expertise. Retention elections thus had the greatest potential really to purge communist judges. Accordingly, they also stirred up the biggest controversies, and faced opposition both from MPs and from new, democratic court presidents. Many feared that retention would negatively fall also on those judges who had carried out their duties justly, but joined the Communist Party as they had no other means of pursuing a judicial career.

Interestingly, the Deputy Prime Minister toned down these fears with the argument that retention was not intended to purge large numbers of rank-and-file judges, as ‘those judges who had been compromised by violation of laws and judicial ethics’^101^ had already left the judiciary. The government also admitted that, given the time required for a large-scale transformation of the judiciary, its hands were tied due to a shortage of judges and the need to start other parts of a legal reform. In the end, apart from in the Federal Supreme Court, retentions did not lead to massive purges in the judicial ranks. The lukewarm political support and opposition from new court presidents sealed the mechanism’s fate. De-communisation of the courts was, therefore, already in the early 1990s, planned as a continuous and meticulous process.^102^ Instead of large-scale dismissals, the government vowed to invest in fostering the quality of legal education, professional exams and the material needs of courts.

The marginal effect of the five mechanisms aimed at judicial purges also showed that they were ill-equipped to tackle the often informal participation of judges in the politicisation of justice and abuses of individual rights. For these reasons, removals of judges due to their loyalty to the communist regime were minimal. To put it bluntly, the Czech post-communist regime simply had no choice but to retain a significant number of judges from the communist era. The only available solution was to re-socialise communist judges, i.e. ‘fill old bottles with new wine’ and produce as many ‘new bottles’ as soon as possible.

Unfortunately, this hope was not realised. Path-dependence prevailed in the end and control of the higher echelons of the judiciary remained in the hands of judges from the communist era. The communist-era judges thus did not lose their influence. On the contrary, even in the early 2020s the presidents of both the Supreme and Supreme Administrative Courts and key figures of the High Court in Prague (the president and two out of the five vice-presidents) were still former members of or candidates for the Communist Party.^103^ The numbers of rank-and-file judges at top courts are also striking. In 2019, on the anniversary of the Velvet Revolution, the updated list published by the Ministry showed that 13.5 per cent of all active members of the judiciary had joined the Communist Party prior to 1989.^104^ Most of the judges with a communist past were sitting in the Supreme Court (37 per cent), the High Court in Olomouc (34 per cent) and the Regional Court in Prague (27 per cent).^105^ In contrast, the percentage of ex-communists among judges of district courts has been relatively low. In 2018, the majority of district courts had a proportion of judges with a communist partisan legacy which was lower than 15 per cent. This ‘inverse pyramid,’ showing a linear relationship between the percentage of ex-communists and level of the judicial career structure,^106^ is of course a natural consequence of the fact that lower-court judges are younger and the personnel change at district courts happened faster as those judges were drawn from the younger population.

In sum, the most effective of the judicial purges was the removal of ex-communist court presidents, due to the risk of their ties with and loyalty to the Communist Party, as well as inadequate moral, ethical or expertise standards. The derived mechanisms of transitional justice prevailed and left aside the core transitional justice issue, the role of judges or court presidents in human rights violations. In a way, replacing court presidents was a necessary top-down measure of the consolidation of democracy. It solved the dilemma of an immediate lack of qualified judges who would not have connection to communist elites. New courts presidents were meant to oversee the commitment of the judiciary to the rule of law, democracy and the protection of individual rights. As mentioned above, ‘new bottles’ were not available in sufficient numbers, and thus new court presidents had to make sure that ‘old bottles are filled with new wine’.

The removal of court presidents also had the broadest support from both the political and judicial ranks. Interestingly, the issue of whether and how much to purge the judiciary did not lead to many disputes among the political elite. Some MPs expressed the fear that the change of judges was not enough or not fast enough, but in general dealing with the past within the judiciary was primarily outsourced to judges themselves. It seems that successful implementation depended on the cost–benefit considerations of politicians. The more successful mechanisms were those directly tailored to the needs of the judiciary and carried out directly by the government. Those mechanisms the implementation of which was left to the judiciary itself mainly failed to deliver substantive change.

## Haunted Judiciaries and Invisible Transitional Justice

The hunger to uncover the past of communist judges never really left the Czech public discourse. In 2011, a human rights activist, Tomáš Pecina, brought an action against the Ministry of Justice for failure to provide him with information about former members of the Communist Party on the bench.^107^ The case travelled all the way to the Constitutional Court, which agreed with the petitioner and argued that there was a public interest in obtaining information on the past roles of judges under the communist regime. When the Ministry of Justice complied and published the list of active ex-communist judges, it caused an outcry both outside and within the judiciary.

However, the length of the time which passed since human rights violations to the regime transition and opening of secret services archives complicated the whole process and triggered questions about the reliability of the information stored in these archives. Judges who were correctly included in the list of active ex-communists downplayed the importance of their membership in the Communist Party, whereas judges whose names appeared in the list by mistake vigorously protested and threatened the Ministry of Justice with defamation actions.^108^ The media started digging up stories about individual judges and their careers. The public was appalled and flooded social media with hate comments directed at the judiciary. Some politicians knew about the communist pasts of a couple of judges, but they too were surprised by the number of ex-Communist Party members still present in the top tiers of the judiciary. This forced ‘coming out’ of ex-communist judges has haunted the Czech judiciary ever since. The communist past of candidates to apex courts occasionally resonated among the public in following years and culminated in 2023 with the resignation of the former ICC vice-president and constitutional court justice candidate, Fremr.

How was this possible if Czechia had five complex mechanisms that addressed almost all aspects of judicial turnover? As we noted in the previous Section, although individual mechanisms seemed comprehensive, they often did not correctly aim at the roles of judges under the communist regime.

1. Core transitional justice mechanisms, such as lustration, but also criminal prosecutions, aimed at judges who broke Czechoslovak communist law, manipulated evidence or helped communist power to orchestrate criminal trials. The majority of these exemplary show trials, however, appeared in the 1950s and judges complicit therein were no longer on the bench in the late 1980. Hence, the long period between crimes and retributive processes significantly impaired the effectiveness of these transitional justice mechanisms.
2. The second targeted group were judges who implemented communist law that was in conflict with human rights principles and international commitments of communist Czechoslovakia. These were judges who sentenced political opponents to prison, prosecuted the signatories of Charter 77 or confiscated property of illegal immigrants. The retribution for their activity was no less complicated. While some of them left the judiciary on a voluntary basis, the Supreme Court was hesitant to proceed with criminal or disciplinary proceedings against the rest of them, arguing that such a step would violate both judicial independence and the principle of non-retroactivity.
3. The derived transitional justice mechanisms against judges who did not commit any crimes per se, but who were ideologically aligned with the Communist Party and communist understanding of judicial independence, or who lacked required expertise or ethical qualities, were even more problematic. Although the early transitional processes removed communist court presidents (who had great formal and informal influence on the rank-and-file judges^109^), later on, removals from ideological or moral reasons also became theoretically and legally challenging because of principles of non-retroactivity and protection of judicial independence. Nowadays, the jurisprudence of the European Court of Human Rights renders political removals (which would have been the essence of a removal of judge appointed legally by the communist power) almost impossible. Unfortunately, lustration, a mechanism European supranational bodies tolerated for a limited period of time despite of its extraordinary interference in public offices, was largely misplaced when it came to the role of judges and vetting procedures.

With certain simplification, we can argue that despite the initial effort, the way how Czechia dealt with the past of the judiciary was not only substantively insufficient, but it also failed to clearly communicate the extent to which the communist-era judges participated on the communist regime and its ideology. Why is it important? It is true that after the transition, political leaders typically enjoy relatively large space for manoeuvre when deciding how to deal with representants of the previous regime, based on normative or pragmatic considerations. However, as Mainwaring and Peréz-Liňán argued, ‘the cumulative experience of past generations affects the level of democracy in contemporary political regime’ and that the justice system plays a central role therein, as it ‘operates as an institutional carrier of regime legacies’.^110^ The Czech story, magnified by the recent resignation of judge Fremr, suggests that the lack of visible dealing with the past of judges may haunt and reduce the legitimacy of the courts for a surprisingly long time.

Moreover, the development in other CEE countries hints that judicial turnover after the democratization is more important than core transitional justice scholarship anticipated. First, the evidence of judges being the vehicles of transitional justice comes from Latin America,^111^ Africa,^112^ South-East Asia^113^ and post-war Europe.^114^ While some scholars note that the pro-transitional justice behaviour of courts emerges as a symbol of judges’ atonement for their role in the oppressive regimes,^115^ others point out that such behaviour is further conditioned by structural reforms^116^ or changes in behavioural patterns, which again depend on transitional justice policies and the successful implantation of new rule-of-law institutions.^117^ The empirical evidence of pre-transition judges behaving leniently towards the prosecution of past crimes prevails.^118^ In the end, the Czech story is similar, as unreformed top courts protected their own colleagues and decided formalistically on transitional justice issues.^119^

Second, the lack of judicial purges and truth-seeking harms the legitimacy of judiciaries in the long run. De-communisation scholars have repeatedly pointed out that lustration that targets broad bureaucratic systems has positive effects on the degree of democratisation.^120^ As with ‘the skeletons in the closet’ argument, judges with an unknown past instigate biases in the public perception of transitional justice implementation.

Third, the unaddressed communist past of judges became an instrumental wild card in the hands of political leaders who sought to tinker with judicial independence.^121^ Many populist leaders have recently adopted the language of de-communisation and cleansing the judiciaries of past non-democratic legacies. Fidesz has long argued that the corruption and inefficiency of the Hungarian judiciary is the result of missing de-communisation.^122^ Orbán initially advocated large-scale reforms and court-packing plans initiated in 2011 using de-communisation rhetoric.^123^ Similarly, the Polish PiS has repeatedly stated that judicial reforms tackle the corruption that results from the Polish judiciary being plagued by remnants of its communist past. Prime Minister Mateusz Morawiecki has repeatedly stressed that the Polish judiciary remained independent of any checks and balances and lacked public accountability due to the form of the 1989 roundtable talks, which allowed the new democratic-era courts to be staffed with communist-era judges.^124^ In both cases, the political statements failed to acknowledge how many judges with a communist past were actually still sitting at their national courts. De-communisation was primarily a slogan to delegitimise the judiciary and to justify problematic judicial reforms.

These three reasons have potential repercussions not only for those countries that are transitioning from a fully non-democratic regime, but for any countries that struggle with low legitimacy of courts and mistrust in the individual independence of judges appointed under previous regimes (often in illegitimate processes). The Czech story has demonstrated that elites need to take the re-establishment of the legitimacy of the judiciary seriously. Both key questions, whether and how to punish judges who participated actively in human rights violations and what to do with judges whose decision-making has always complied with principles of the rule of law, but who were otherwise ideologically aligned with the previous non-democratic elite, indoctrinated, or illegitimately appointed, need to be addressed as swiftly as possible, and in a transparent open discussion that would be comprehensible and visible also to the broader public.

The strategic use of an anti-communist narrative more than three decades after the fall of the Berlin Wall has responded to societal sentiments. The lack of judicial purges failed not only transitional justice, but also the rebuilding of the rule of law. As demonstrated by the Czech case, judicial purges in post-communist countries had to follow several objectives. Apart from cleansing the judiciary of judges who had taken part in crimes of the communist regimes, they were also aimed at reinstating mental judicial independence,^125^ increasing the prestige of judges and instilling new ethical and moral standards into the judiciary. As we have seen in many CEE states, the lack of mental independence of communist judges, who were used to exist in very hierarchical models with loyalty and dependence of rank-and-file judges on their senior peers and court presidents, prevented the internal reforms and democratization of judiciaries from taking place. Instead, judiciaries started to replicate the old patterns.^126^ The people did not like it and skilful politicians managed to exploit this anger towards judges. This, in our understanding, demonstrates that a policy as fragile and, at the same time, politically salient as transitional justice in the judiciary needs to be comprehensible and visible to the public. Otherwise, it may easily become a hostage to political elites pursuing their own interests at the expense of judicial independence.

## Conclusion

In the last decade, the notion of judicial vetting and lustration has changed. Both mechanisms have abandoned their exclusive relationship with regime transition and are now more broadly invoked as extraordinary tools that may be applicable in situations where the legitimacy of the judiciary, its commitment to the rule of law, independence or justice is in question. Lustration of judges has turned out to be a rather standard, even internationally approved, vetting mechanism for dealing with wide-spread judicial corruption scandals.

In this article we have offered a historical analysis of the Czech post-1989 judicial turnover. We have argued that the Czech case contains several interesting observations for the ongoing debates on judicial vetting in Poland, but also for eventual future reforms to come in countries like Hungary, Romania, Ukraine, Albania, Georgia, Kosovo and Macedonia.

First, the public cares about the role of judges under the previous regime. This means that political elites need to take the transitional removals and judicial turnovers very seriously. Second, timing is of the essence, both from the perspective of access to original documentation, but also from the point of view of supranational organisations that typically are willing to accept the transitional rule of law (and processes like lustration) for only a limited time. Third, mechanisms of transitional justice do get into significant tension with principles of judicial independence, non-retroactivity and the rule of law.^127^ In the most extreme case, they can even lead to the erosion of judicial independence and the separation of powers, which would set a dangerous precedent for future political interferences. Large-scale judicial turnover may negatively impact on the quality and effectiveness of judicial decision-making. Many young transitional countries struggle with a shortage of judges unburdened by the past and must strike a compromise between qualified candidates and judicial candidates with no ties to the past regime.^128^ This, in turn, leads to an unfortunate trade-off between the effectiveness of justice and judicial independence.

We have shown that Czechia, despite having the harshest lustration law in the region and mechanisms that ought to have address the individual accountability of judges who participated on communist regime crimes, who violated standards of international law, or lacked knowledge or ethical standards, has eventually managed to achieve only limited de-communisation of the judiciary. In fact, of the five transitional justice mechanisms aimed at purging the judicial ranks, only one—dismissal of communist-era court presidents—has proved to be a fully successful strategy. The other four mechanisms—the retention, lustration, disciplining and criminal prosecution of judges—have yielded limited results, albeit for different reasons.

At the same time, the Czech case also shows that typical transitional justice mechanisms may not be well equipped to resolve the problem of the complicity of judges in past crimes for three reasons. First, judges played different roles in past crimes from political elites, and the traditional transitional justice mechanisms usually fail to capture the specific judicial complicity with the non-democratic regime. Second, the principles of the separation of powers and judicial independence, vigorously defended by judges themselves, preclude judicial reform from being easy. Third, practical exigencies such as the shortage of educated lawyers not tainted by cooperation with a previous regime further limit the effects of purges and the vetting of judges in transitional countries.

Yet addressing the authoritarian and totalitarian past of judges is extremely important for three reasons. First, judges played an important role in the implementation of transitional justice policies, and there is growing evidence that judges who participated in a totalitarian or authoritarian regime are biased against transitional justice and unlikely to implement its mechanism effectively. Second, the lack of judicial purges and truth-seeking harms the legitimacy of the judiciary and public confidence in the courts. Moreover, it prevents the mental transition of judges who may adopt a skewed understanding of judicial independence, who block the development of ethical standards and engage in problematic behaviour on as well as off the bench. Several countries in CEE show clear signs of these problems. In Ukraine and Slovakia limited judicial purges allowed informal corruption networks around judges to emerge and survive the regime change.^129^ It is also becoming clear that these judiciaries will not cleanse themselves due to the corporativist career-model and replication of older patterns.

Third, the failure to deal with the past of judges entails a risk that political leaders who wish to tinker with judiciaries will use the randomly uncovered stories as a wild card to delegitimise the judiciary and to justify the interference with its independence. Skeletons in the closet then start haunting judges. We have seen this strategy applied over and over again by populist governments in Hungary, Poland and Romania. These politicians simply abuse belated lustration and other mechanisms in order to get rid of their opponents on the bench and staff the courts with ideologically aligned judges.

The clashes between these dilemmas thus place judicial purges between the proverbial rock and hard place. It is clear that the repercussions of insufficient reckoning with the pasts of judges transcend transitional justice. Even more importantly, the events of the last decade have demonstrated that judicial purges are still a highly relevant topic for European countries. For example, in order to address widespread judicial corruption and low trust in courts, Albania implemented judicial lustration in 2017 and Ukraine removed all court presidents in 2014. Despite the (relatively surprising) support of European supranational bodies, both lustration processes brought mixed results. Ukraine in particular demonstrates how influential informal institutions are in sabotaging structural reforms: Ukraine left the re-election of court presidents, removed due to corruption, bossing and patronage, to rank-and-file judges. At majority of courts, judges re-elected those court presidents that were previously removed by the lustration law.^130^

Retention and lustration of judges is very much discussed also by the scholarship exploring what to do with judges artificially packed into courts by populist and non-democratic leaders. In the case of post-Kaczyński’s Poland, scholars have already suggested several ways of instigating criminal prosecutions of individual judges who have acted against the spirit and letter of international and EU law,^131^ as well as the potential retention and vetting of all judges illegitimately selected as a part of PiS court-packing policies.^132^ It is, however, still worth stressing that, compared to other political elites, judges, including those selected under a non-democratic government, are protected by internationally entrenched principle of judicial independence, which is also recognised as a building block of the rule of law doctrine. Although European supranational bodies are slowly becoming more receptive towards the idea of lustration as an extraordinary mechanism implemented when other means fail, the shortcomings of the mechanism demonstrated in the Czech case suggest that much more work on the relationship between transitional justice measures and the transitional dimension of judicial independence is needed.

## Footnotes

1 Apart from his position at ICC, Fremr also served as a judge at the International Criminal Tribunal for Rwanda.

2 Procházková (2023).

3 USTR (2023), https://www.ustrcr.cz/wp-content/uploads/2023/10/Zprava-Fremr-USTR-pro-KPR.pdf.

4 Skapska (2003, p. 199, p. 202).

5 Robertson (2006, p. 73, p. 87).

6 Schwartz (1994, p. 141, p. 142).

7 Kuhn (2011), Kosař (2016, 2017).

8 Holgado and Urribarri (2023), Keck (2023). ETC.

9 Szwed (2023).

10 Kosař and Šipulová (2023a, b).

11 Teitel (2014), similarly Sajo — militant rule of law concept.

12 Bobek et al (2023), Szwed (2023) and Přibáň (2023).

13 Šipulová and Smekal (2021).

14 Szwed (2023).

15 Kritz (1995), Teitel (2002) and Elster (2004).

16 Huntington (1993); Czarnota et al. (2005); Calhoun (2004), Cohen (1995), David (2015), Filjakowski (2014), Grodsky (2009), Kornai & Rose-Ackerman (2004), Stan (2009), Nalepa (2010), Teitel (1997) and Stan & Nedelsky (2015).

17 David (2011), Horne (2004), Huntington (1993); Iancu (2010), Moran (1994), Nedelsky (2004) and Szczerbiak (2002).

18 Ledeneva (2008), Popova (2012).

19 Kűhn (2011, p. 163).

20 Šipulová & Smekal (2021), Dyzenhaus (2003b) and Kavanagh (2023).

21 Šipulová & Smekal (2021).

22 Ocantos (2014), Huneeus (2010), Skaar (2011) and Hilbink (2007).

23 Arendt (1963, p. 25). Arendt states that in the early 1960s, approx. 5000 out of 11,000 judges had served in Nazi courts. The purging took place only in 1962, possibly in relation to the publicity the Eichmann trial gave to the missing vetting of the German public administration. Arendt uses the composition of courts to explain why post-war German courts proceeding in criminal proceedings involving Nazi criminals very leniently.

24 Huneeus (2010).

25 Skaar (2011), Hilbink (2007) and Graver (2018).

26 Iancu (2021).

27 See Bobek (2008), Kosař (2016), Kühn (2011, 2021), Čuroš (2021). On the hierarchical model of officialdom in general see Damaška (1986, pp. 16–46 and 181–239).

28 Vyshinsky (1948, pp. 303ff).

29 See Toharia (1975, p. 475), and Hilbink (2008).

30 It abolished the Constitutional Court, the Supreme Administrative Court and the Election Court.

31 Kosař (2016) and Kühn (2011).

32 Probably the most famous show trial was the prosecution of Milada Horáková, a female opposition leader who in 1950 was sentenced to death on fabricated charges of conspiracy and treason. See below notes 68 and 69.

33 Kühn (2021).

34 These bodies worked at the regional level, composed of the regional secretary of the Communist party, the bursar and three other regional chiefs of state security. They misused the rhetoric of communist security in order to monitor the activity of the state prosecution and prepare important politicalised trials.

35 See Ulč (1972); Vorel et al. (2003); and Kühn (2011, 2021).

36 See Wagnerová (2003, pp. 163–179).

37 By retention we mean a periodic process the aim of which is to decide whether a judge should stay in office (see Kosař 2016, pp. 78–80).

38 See Motejl (2009, pp. 813–821. p. 821).

39 Ibid.

40 Czech Social Science Data Archive of the Czech Institute of Sociology.

41 See Bobek (2008, p. 118) and Čuroš (2021).

42 Szwed (2023).

43 This question in particular is discussed in context of Poland, with both European courts labelling the packed judges as „non-judges”. See Szwed (2023), Leloup (2022, 2023), Leloup and Kosař (2022).

44 Elster (2003); Teitel (2002).

45 Law on courts and judges. Transcription from meeting no. 16, 8 July 1991.

46 Virtual Library of the Chamber of Deputies, Parliament of the Czech Republic, transcription of meeting. 4, 10 July 1990.

47 Czech Social Science Data Archive of the Czech Institute of Sociology.

48 Kühn (2011).

49 Kosař and Šipulová (2023a, b).

50 Kühn (2011, p. 163).

51 Wagnerová (2003, p. 169).

52 Bröstl (2003, 143).

53 Wagnerová (2003, p. 169). See also Virtual Library of the Chamber of Deputies, Parliament of the Czech Republic, transcribed record of 30 March 1990 meeting, http://www.psp.cz/eknih/1986cnr/stenprot/025schuz/s025027.htm.

54 Virtual Library of the Chamber of Deputies, Parliament of the Czech Republic, draft proposal of the amendment of Act 335/1991 on courts and judges, http://www.psp.cz/eknih/1992fs/tisky/t0040_00.htm.

55 For further details see Blankenburg (1995, p. 223).

56 Ibid., pp. 240–241. See also Markovits, pp. 2270, 2271–2272.

57 Note that some judges active in 1989 did not obtain standard legal education as they were alumni of the so-called ‘crash courses for the working class’ (that usually took only one year). These ‘crash courses’ were introduced by the Communist Party in the periods of shortage of loyal judges with a standard education (namely after the communist coup d’état in 1948 and after the purges in the wake of the crushing of the 1968 Prague Spring). Judges who had been through these ‘crash courses’ were generally considered incompetent hard-core communists.

58 See judgment of the Czech Constitutional Court No. IV. ÚS 23/05 of 17 July 2007 (N 111/46 SbNU 41), paras 21 and 48.

59 Nalepa (2010, p. 65).

60 Orbman, (1991, pp. 4–11) and David (2011).

61 There was also a Small Lustration Law (Law No. 279/1992) dealing with members of police forces.

62 Law No. 451/1991 Coll., on Standards Required for Holding Specific Positions in the State Administration of the Czech and Slovak Federal Republic, Czech Republic and Slovak Republic (hereinafter ‘Large Lustration Law’).

63 Parliamentary Assembly of the Council of Europe (1996). International Labour (1992). Quoted from Přibáň. (2017, p. 189).

64 Kosař (2008).

65 The very first proposal of the Large Lustration Law was nevertheless even harsher, and was criticised by Havel and some other dissidents as draconian and a basis for a witch hunt, with limited application of the fair trial principle.

66 David (2011).

67 Gillis (1999, p. 56).

68 Art. 1 (1) of the Large Lustration Law.

69 Cf. Nalepa (2010, p. 68).

70 Czechoslovak Constitutional Court, judgment Pl. ÚS 1/92 of 26 November 1992 and Czech Constitutional Court, judgment Pl. ÚS 9/01 of 5 December 2001.

71 See Communication of the President of the Czech Supreme Court, Antonín Mokrý, with the Ministry of Justice, 23 April 1992, no. ÚPP-190/92.

72 See ECtHR, Polednová v the Czech Republic, decision of 21 June 2011, 2615/10. See also note 47 above.

73 See the Czech Supreme Court judgment of 4 February 2008, building on its previous judgment no. 7 Tz 179/99 of 7 December 1999.

74 Virtual Library of the Chamber of Deputies, Parliament of the Czech Republic, transcription of meeting no 28, 17 December 1991.

75 Piana (2010) at 132.

76 Ibid., p. 130.

77 Ibid., p. 99.

78 Spáč (2014).

79 Piana (2010) at p. 98.

80 Piana (2010) at 98–100, 108 and 110.

81 Wagnerová (2003). See also Kühn (2011), p. 181.

82 See e.g. Kühn (2011), p. 181; Bobek, (2008) at pp. 99, 118–119; and Kosař (2016)

83 This classification builds on our own content analysis of parliamentary debates on all transitional justice mechanisms as well as judicial reforms.

84 See Sect. 2 of this article, as well as e.g. Kühn (2011).

85 Virtual Library of the Chamber of Deputies, Parliament of the Czech Republic, transcription of meeting discussing Lustration, no. 11, 8 January 1991.

86 Ibid.

87 Virtual Library of the Chamber of Deputies, Parliament of the Czech Republic, transcription of meeting no. 9 on the election of judges to the military division of the Federal Czechoslovak Supreme Court, 28 November 1990.

88 Virtual Library of the Chamber of Deputies, Parliament of the Czech Republic, transcription of meeting discussing the execution of lustration, no. 11, 8 January 1991.

89 Šipulová and Smekal (2021) and Bröstl (2003, pp. 141–159).

90 Šipulová & Smekal (2021).

91 See e.g. Prague Municipal Court, decision 37 C 116/2005 of 16 November 2005 against a High Court of Olomouc judge who had been listed in secret police archives as a potential collaborator. The Municipal Court confirmed the Ministry of the Interior’s obligation to justify the refusal.

92 Virtual Library of the Chamber of Deputies, Parliament of the Czech Republic, transcription of meeting no. 16 discussing Law on courts and judges, 8–9 July 1991 and meeting no. 11, discussing lustration, 8 January 1991, meeting no 21, report on the tasks of the Czech judiciary, 10 July 1991.

93 Virtual Library of the Chamber of Deputies, Parliament of the Czech Republic, transcription of meeting no 28, 17 December 1991.

94 See Bröstl (2003, p. 143), Kühn (2011, p. 124), Bobek (2015) and Kosař (2016).

95 Note that judges dismissed from positions as court presidents often remained in the judiciary as rank-and-file judges.

96 On the importance of court presidents in the post-Velvet Czech judiciary see Bobek (2010), Kosař (2016) and Kosař (2017).

97 Virtual Library of the Chamber of Deputies, Parliament of the Czech Republic, transcription of meeting no. 16 discussing Law on courts and judges, 8–9 July 1991.

98 The Supreme Court of the Czech Republic, S Kno 1/94, decision of 31 August 1994.

99 Virtual Library of the Chamber of Deputies, Parliament of the Czech Republic, transcription of meeting no 21, report on the tasks of the Czech judiciary, 10 July 1991.

100 Ibid.

101 Virtual Library of the Chamber of Deputies, Parliament of the Czech Republic, transcription of meeting no. 16 on the Law on courts and judges, 8 July 1991.

102 Virtual Library of the Chamber of Deputies, Parliament of the Czech Republic, transcription of meeting no. 16 on the amendment of the Constitution of Czechoslovak Federal Republic, 16 July 1991.

103 Recall again that all Czech judges had to undergo lustration, but mere membership of the Communist Party of Czechoslovakia is not an obstacle to holding judicial office according to the Large Lustration Law.

104 Ministry of Justice, list of judges—former members of the Communist Party of Czechoslovakia, first published on 7 January 2011, updated on 17 November 2019. Available at https://www.justice.cz/web/msp/clenstvi-v-ksc1.

105 Ibid and iRozhlas, 17 November 2018, available at: https://www.irozhlas.cz/zpravy-domov/soudci-clenstvi-v-ksc_1811170600_pek.

106 Kühn (2011, p. 181).

107 By the 1980s, the majority of judges were automatically also members of the Communist Party, therefore, democratic politicians of 1989 were willing to accept the past membership as a mere formality, and not a signal of ideological affiliation with the Communist Party. The request was based on the Law No. 106/1999 Coll., on Free Access to Information (Freedom of Information Law).

108 See e.g. Lidové noviny (2011) and Mladá fronta DNES (2011).

109 Blisa and Kosař (2018); Kosař and Šipulová (2024).

110 Mainwaring and Peréz-Liňán (2013, p. 394).

111 Ocantos Gonzalez (2014).

112 Yusuf (2008, 2013).

113 Holliday (2014), Bedner (2013) and Mochizuki (2017).

114 Arendt (1963).

115 Hunneus (2010).

116 Skaar (2011) and Hilbink (2007).

117 Ocantos Gonzales (2014).

118 Yusuf (2008) and Spáč (2020).

119 Šipulová & Smekal (2021).

120 Horne & Levi (2004).

121 Iancu (2021).

122 Hungary Today (2018).

123 Kosař and Šipulová (2020).

124 Chhor (2018) and Morawiecki (2017).

125 Čuroš (2021) and Šipulová and Spáč (2024).

126 Popova & Beers (2020).

127 Dyzenhaus (2003a).

128 Ellett (2015), Kosař (2016), Betts et al. (2023), Oko (2005), Dyzenhaus (2003a) and Yusuf (2008).

129 Popova (2020) and Spáč (2020).

130 Popova (2020).

131 Von Bogdandy and Spieker (2023).

132 Kosař and Šipulová (2023a, b).
